# The Role of IL-36 in Infectious Diseases: Potential Target for COVID-19?

**DOI:** 10.3389/fimmu.2021.662266

**Published:** 2021-05-13

**Authors:** Xiaofang Wang, Panpan Yi, Yuejin Liang

**Affiliations:** ^1^ Department of Infectious Diseases, Key Laboratory of Viral Hepatitis of Hunan, Xiangya Hospital, Central South University, Changsha, China; ^2^ Department of Microbiology and Immunology, University of Texas Medical Branch, Galveston, TX, United States; ^3^ Institute for Human Infections and Immunity, University of Texas Medical Branch, Galveston, TX, United States

**Keywords:** IL-36, IL-1 family, cytokine, infection, COVID-19

## Abstract

IL-36 is a member of the interleukin 1 cytokine family, which is currently experiencing a renaissance due to the growing understanding of its context-dependent roles and advances in our understanding of the inflammatory response. The immunological role of IL-36 has revealed its profound and indispensable functional roles in psoriasis, as well as in several inflammatory diseases, including inflammatory bowel disease (IBD), systemic lupus erythematosus, rheumatoid arthritis (RA) and cancer. More recently, an increasing body of evidence suggests that IL-36 plays a crucial role in viral, bacterial and fungal infections. There is a growing interest as to whether IL-36 contributes to host protective immune responses against infection as well as the potential implications of IL-36 for the development of new therapeutic strategies. In this review, we summarize the recent progress in understanding cellular expression, regulatory mechanisms and biological roles of IL-36 in infectious diseases, which suggest more specific strategies to maneuver IL-36 as a diagnostic or therapeutic target, especially in COVID-19.

## Introduction

Interleukin (IL)-36 is a member of the IL-1 cytokine family. It plays a role in the orchestration of innate and adaptive immunity and appears to have pro-inflammatory activities (1, 2). The IL-36 family includes three agonist ligands (IL-36α, β and γ, previously known as IL-1F6, IL-1F8, IL-1F9), which bind to heterodimeric receptor complexes, the IL-36 receptor (IL-36R, also known as IL-1Rrp2) and co-receptor IL-1 receptor accessory protein (IL-1RAcP) (3). The IL-36 receptor antagonist (IL-36Ra, formerly known as IL-1F5), an antagonist in the IL-36 family, inhibit IL-36-induced inflammation *via* competing with IL-36 receptor (4). The pro-inflammatory role of IL-36 is well studied in psoriasis (5–9), inflammatory bowel disease (IBD), systemic lupus erythematosus (SLE), rheumatoid arthritis (RA) and cancer (5, 10–14). IL-36γ is a potential diagnostic marker of psoriatic inflammation (15). The success of treatment using a monoclonal antibody against IL-36 receptor in generalized pustular psoriasis patients highlights the promising potential strategy of blocking the IL-36/IL-36R signaling pathway in clinical therapy (7). IL-38 (previously known as IL-1F10), which shows the highest similarity of percentage amino acid identify with IL-1Ra and IL-36Ra, may act as an IL-36R antagonist (16, 17). Recent study demonstrated the inhibitory function of IL-38 on the phosphorylation of P38 MAPK and the subunit P65 of NF-κB induced by IL-36γ in human keratinocytes and endothelial cells (18). Besides, IL-38 was released from apoptotic cells and restricted human macrophage-dependent induction of IL-17 (19). IL-38 knockout mice had delayed disease resolution with exacerbated IL-17-mediated inflammation, which is reversed by the administration of matured IL-38 in a mouse model of psoriasis (20). Hence, IL-38 is considered an anti-inflammatory factor in the pathologies of autoimmune diseases.

Accumulating evidence suggests that IL-36 is also involved in infectious diseases, especially viral and bacterial infections. Using knockout mice for either IL-36 cytokines or receptor, researchers have revealed that IL-36 plays both protective and pathological roles in distinct animal models of infection (21–23). On one hand, IL-36 is beneficial for pathogen clearance by promoting protective immune responses (21). On the other hand, IL-36 amplifies inflammatory responses, leading to excessive immune infiltration and tissue damage (24). In this review, we first focus on the cellular source and target cells of IL-36, and then highlight the recent advances of the IL-36 research in infectious diseases. At the end, we discuss IL-36 as a potential therapeutic target for COVID-19.

## Processing of IL-36 and Downstream Signaling Pathways

Similar to other members of IL-1 family, the inactive precursors of IL-36 require proteolytic and post-translational processing for their maturation and pro-inflammatory activity, respectively (25, 26). Neutrophil granule-derived proteases cathepsin G (Cat G), elastase and proteinase-3 are involved in the processing (25–30). IL-36α is processed and activated by Cat G and elastase respectively *via* truncating at alanine 4 and lysine 3. IL-36β is selectively stimulated by Cat G through its cleavage at residue arginine 5 (28). IL-36γ can be activated by elastase or proteinase-3 by means of cleavage at the residue valine 15 (28). In addition, IL-36γ also can be cleaved between residues glutamic acid 17 and serine 18 by Cathepsin S (29). Removal of a small number of residues from the N termini of IL-36 increases the biological activity by more than 10,000-fold (26). Similarly, IL-36Ra is cleaved to become mature form by elastase through removal of its N-terminal methionine (30), and the matured IL-36Ra competes with IL-36 cytokines for IL-36 receptor binding to suppress IL-36 activity (26).

Binding of IL-36 agonists (IL-36α, -β and -γ) to IL-36R/IL-1RAcP heterodimer induces inflammatory mediators through MyD88-, MAPK- and NF-κB-dependent signaling pathways (31–33). It is demonstrated that *Staphylococcus aureus* (*S. aureus*) exposure drives murine skin inflammation, which is caused by the IL-36R/MyD88-mediated IL-17 (34). IL-36γ stimulation also promoted the expression of NF-κB target genes (TNFAIP3, NFKBIA, NFKB2, CXCL8, and BIRC3) in a MyD88-depenent manner in human epidermal keratinocytes (35). Besides, IL-36 α employed NF-κB and STAT3 for IκBζ induction, and induced several psoriasis-related cytokines and chemokines in psoriatic skin (32). Additionally, activation of IL-36/IL-36R axis enhanced the secretion of IL-6, IL-8, and granulocyte-macrophage colony-stimulating factor by activation of Erk1/2, MAPK and JNK (3), while IL-36Ra suppressed the IL-36 agonist-triggered IL-8 expression (26).

## Cellular Source and Function of IL-36

At steady-state, IL-36 is mainly expressed in epithelial cells and fibroblasts (36–41). Several kinds of cells, including epithelial cells, mouse T cells and myeloid cells can respond to IL-36 stimuli (37, 38, 41–43).

### Epithelial Cells

IL-36 was predominantly expressed in epithelial cells in experimental colitis, allergic lung inflammation, chronic rhinosinusitis and influenza A virus infection (22, 36, 41, 44, 45) and was upregulated by proinflammatory cytokines, such as IL-17 (41, 46). Reproductive tract epithelial cells also increased IL-36γ and IL-36R expression following treatment with microbial products (47). It is notable that epithelial IL-36 could be expressed as a full-length form and required a cleavage to the biologically active form (41). Epithelial cells can secret inflammatory cytokines in response to IL-36. Subcutaneous injection of IL-36α induced various inflammatory factors including IL-17, IL-20, IL-22, IL-23, interferon (IFN)-γ, TNF-α and KCs (48). IL-17 production by Th17 cells may upregulate all three IL-36 expression from human keratinocytes, creating a feedback loop that drives inflammation and disease development (49). Human keratinocytes were potent sources of chemokines following the exposure of IL-36 cytokines, leading to the recruitment of macrophages, T cells, and neutrophils (43). Notably, human keratinocytes upregulated type I and II IFN-responsive genes in response to IL-36, leading to potent cytokine production (35, 43). These findings indicate that IL-36 is critical for the early regulation of IFN and immune cell recruitment in the skin (50–52). Expression of IL-36R by skin-resident cells (e.g., keratinocytes and fibroblasts), but not the hematopoietic cells (e.g., T cells and DCs) is pivotal for the cutaneous pathology (51). Consistently, using a conditional knockout murine model, Goldstein et al. demonstrated that IL-36R signaling in keratinocytes played a major role in the induction of psoriasis-like dermatitis (52).

### Myeloid Cells

Activated neutrophils were considered a source of IL-36 in various diseases such as experimental autoimmune encephalomyelitis (EAE), chronic rhinosinusitis and influenza infection (21, 41, 53), while neutrophils from naïve mice express low levels of IL-36γ (53). Importantly, IL-36R is abundant on murine neutrophils derived from bone marrow, spinal cord and spleen (38, 53). However, IL-36R was not detectable in blood neutrophils in both mice and patients with inflammatory diseases (41, 53). Consistently, healthy human blood neutrophils failed to express IL-36R and did not respond to IL-36 cytokines (43, 50). Interestingly, IL-36R expression on human peripheral neutrophils could be induced by IL-1β, IL-6, and Der p1 (41), suggesting that both IL-36 cytokines and receptor might be inducible on neutrophils by the local inflammatory milieu. However, a study also reported that IL-36-triggered human bronchial epithelial cell can release neutrophil-associated chemokines such as CXCL8, and promote infiltration, activation, and inflammatory activity of neutrophils (54). In addition, IL-36 may active neutrophils and amplify lung inflammation in mice (55)

Dermal macrophages expressed high amounts of IL-36R transcript (37), indicating that the expression of IL-36R might be associated with its anatomical localization and immune microenvironment. IL-36β was as potent as IL-1β in stimulating human M2 macrophages, but not M1 and dermal macrophages (37). In addition, both human M1 macrophages and mouse lung macrophages were reported to produce IL-36 ligands following bacterial infection and LPS exposure (5, 24), indicating that macrophages might be the source of IL-36 similar to IL-1β and IL-33. Bone marrow-derived macrophages have undetectable levels or express much lower IL-36R compared to DCs (38, 41). Both human and mouse DCs were found to express IL-36R and become activated by IL-36 agonists stimulation (41, 43, 50, 56). Human monocyte-derived DCs expressed 6-fold more IL-36R mRNA than their monocyte precursors and accelerated maturation by IL-36α, β and γ (43, 56). IL-36R was also detectable in human Langerhans cells, which responded strongly to IL-36β stimulation (37). Additionally, plasmacytoid DCs (pDCs) can highly express IL-36R (50, 56). These pDCs bound by IL-36 potentiated Toll-like Receptor (TLR)-9 activation and IFN-α production (50).

### Lymphocytes

T cells are most likely not the main source of IL-36 cytokines, but can respond to IL-36 due to their expression of IL-36R. Unlike the receptors of other IL-1 family members, such as IL-33R and IL-1R, whose expression are upregulated during T cell activation, IL-36R expression is detectable on naïve T cells, but is negligible in differentiated Th cells (14, 57). It is reported that IL-36γ synergized with IL-12 to facilitate Th1 differentiation, but suppressed Th17 differentiation *in vitro* in murine experiments (36, 38). Interestingly, IL-36γ inhibited Foxp3-expression in murine regulatory T cell development through the IL-36R/MyD88/NF-κBp50 axis, while concomitantly promoted the differentiation of Th9 and Th22 cells (58, 59). Therefore, IL-36 plays a critical role in mouse T cell differentiation.

Whether IL-36 have effect on human T cells is still unclear. It is reported that IL-36 may induced IFN-γ production in human CD3^+^ lymphocytes *in vitro* (14, 56). Penha et al. found the IL-36R expression on CD4^+^ T cells in the human blood and intestines, and IL-36β stimulation promoted CD4^+^ T cell proliferation *in vitro* (42). On the contrary, other researchers reported that IL-36R transcripts were undetectable in blood CD4^+^ T cells from healthy donors, and IL-36 failed to effect on resting or activated human T cells (43). Similarly, no obvious colocalization of IL-36R with human T cells in nasal polyps (41). Further study is needed to elucidate the regulation of IL-36R as well as the role of IL-36 in human T cell activation and differentiation. Similar to mouse CD4^+^ T cells, mouse effector CD8^+^ T cells increased IFN-γ production by IL-36γ stimuli (14, 60), and this process required IL-12 or IL-2 synergy (60). In addition, IL-36γ promoted IFN-γ production *in vitro* by murine NK cells and γδ T cells, which were able to express IL-36R (14). IL-36R mRNA was undetectable in mouse B cells in a previous study (38); Resident B cells and plasma cells in inflamed human tissues were found to express IL-36α (61). CD138^+^ and CD79α^+^ plasma cells were identified as the cellular sources of IL-36α in the synovial tissues and psoriatic skin in patients, respectively (62). How IL-36 regulates B cell functions is still not understood.

### Other Cell Types

IL-36R mRNA has been detected in mouse astrocytes and microglia in the brain, but not in primary neurons (39, 40). However, IL-36 cytokines were dispensable for microglia activation and disease development of EAE (39, 40, 53). Increased IL-36α γ expression was also observed in murine hepatocytes following IL-1β/TNF-α/IFN-γ stimulation (12, 63), indicating that IL-36 may play a role in liver diseases. In addition, IL-36β has a pro-inflammatory effect on human synovial fibroblasts and articular chondrocytes in RA, suggesting the potential role of IL-36 in inflammatory responses of autoimmune diseases (64).

## IL-36 in Infectious Diseases

**Figure 1:**
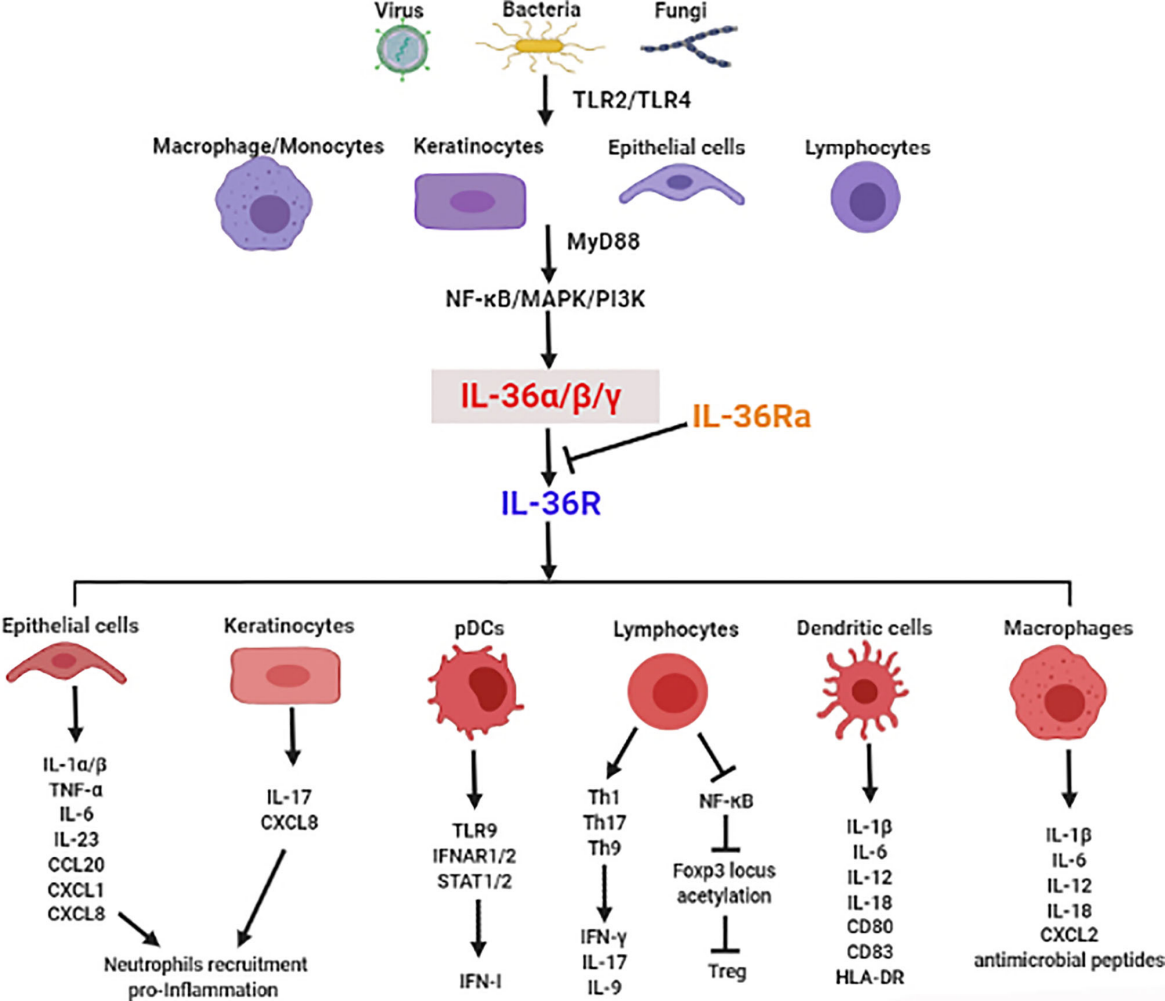
The crucial role of IL-36 in induction of inflammatory cytokines, recruitment of immune cells, modulation of immune cell activation and differentiation, and maintenance of mucosal integrity and barrier function in infectious diseases.

There is mounting evidence for the crucial role of IL-36 in infectious diseases *via* regulation of type I IFN, induction of inflammatory cytokines, recruitment of immune cells, modulation of immune cell activation and differentiation, and maintenance of mucosal integrity and barrier function (**Figure 1**). In this section, we focus on the functional roles of IL-36 in various infectious diseases (**Table 1**).

**Table 1:** The functional role of IL-36 in infectious diseases.

Pathogen	Models or treatment	Experimental results and conclusion	References
HSV-1	IL-36β-/- mice	Increased mortality and weight loss;More severe skin lesions;Similar viral replication	(65)
HSV-2	Exogenous IL-36γ	Increased survival;Delayed disease onset and decreases disease severity;Diminished HSV-2 replication;Induction of the chemokines CCL20 and KC	(66, 67)
Influenza virus (Influenza A/Puerto Rico/8/34 virus)	IL-36R-/- mice	Decreased mortality, but no change of body weight loss;Attenuated lung injury;Higher viral burden;Reduced neutrophils and monocytes/macrophages in BAL fluid	(22)
Influenza virus (influenza A/HK-x31)	IL-36γ-/- mice	Increased mortality and weight loss;Higher viral burden;Increased IFN-β and IL-6	(21)
*Zika*	Designed DNA-encoded IL-36γ	Increased survival rate and less weight loss;Increased IFN-γ and TNF-α expression	(68)
Staphylococcus aureus	IL-36R-/- mice andIL-36R neutralizing Ab	Reduced skin inflammation, decreased disease scores and epidermal thickness;Comparable bacterial loads;Reduced neutrophil infiltration and impaired IL-17 and IL-22 responses	(34, 69)
Pseudomonas aeruginosa	IL-36R-/- and IL-36γ-/- micebut not IL-36α-/- mice	Increased survival;Higher bacterial clearance and reduced bacterial dissemination;Reduced TNF-α, IL-6 and IL-10 expression	(24)
Pseudomonas aeruginosa	Exogenous IL-36γ	Alleviated keratitis;Killed and/or inhibited bacteria growth;Increased β-defensin 3, S100A9 and CXCL10	(70)
Streptococcus pneumoniae	IL-36γ-/- mice andAnti-IL-36γ Ab	Increased mortality;Impaired lung bacterial clearance and increased dissemination;Reduced expression of type-1 and IL-17 cytokines	(23)
Klebsiella pneumoniae	IL-36γ-/- mice andAnti-IL-36γ Ab	Impaired lung bacterial clearance and increased dissemination;Less IL-12, IL-23, and IFN-γ production	(23)
Mycobacterium tuberculosis	Exogenous IL-36γ	Inhibited intracellular survival;Induction of WNT5A expression and autophagy	(71)
Mycobacterium tuberculosis	IL-36R-/- mice	No alteration of survival and body weight loss;No alteration of bacterial burdens;Reduced inflammatory cytokine CXCL1, CXCL2, and IL-6	(72)
Legionella pneumophila	IL-36R-/- mice,but not IL-36α-/-and -γ-/- mice	Increased mortality;Delayed lung bacterial clearance and increased bacterial dissemination; Reduced alveolar macrophage activation and decreased CXCL2/MIP-2 levels	(73)
Citrobacter rodentium	IL-36R-/- mice	No alteration of body weight and clinical signs of inflammation;Increased bacterial colonization;Reduced KC, MPO and inflammatory cell (CD11b+F4/80+Gr-1+) recruitment;Increased Th17, but decreased Th1 and Treg cell associated cytokines	(36)
Candida albicans	IL-36R-/- mice	Greater weight loss;Higher fungal loads;No alteration of IL-17 and IL-22, but decreased IL-23 expression	(74)

### Skin and Mucosal Barriers

IL-36 was first identified as an inducible inflammatory cytokine in mouse keratinocytes following herpes simplex virus type 1 (HSV-1) infection (75). IL-36β-deficient mice developed more severe secondary zosteriform lesions and succumbed more frequently to HSV-1 infection (65). IL-36γ treatment protected mice from lethal intravaginal challenge, as evidenced by limited vaginal viral replication, delayed disease onset, decreased disease severity, and significantly increased survival (66). Further analysis demonstrated that IL-36β promoted type I IFN production through upregulation of IFN-α receptor expression and activation of the STAT signaling pathway in animal model (76). Indeed, IL-36 also promoted type I IFN in IL-36R^+^ pDC (50). Therefore, these studies indicate that IL-36 plays a critical role in innate immunity by boosting type I IFN signaling, inducing pro-inflammatory cytokines, and attracting innate immune cells, such as neutrophils.

Using a murine epicutaneous infection model, Nakagawa and Liu et al. found that *S. aureus* induced IL-1 and IL-36α from keratinocytes *via* secretion of *S. aureus*-expressed phenol-soluble modulin α, leading to the induction of IL-17 and recruitment of neutrophils in the skin (34, 69). Interestingly, IL-36α may not only regulate Th17 cell activity, but also modulate IL-17-production by γδ T cells and type 3 innate lymphoid cells (ILC3) (34, 69). Skin inflammation was dependent on IL-1R and IL-36R signals as well as their signaling adaptor MyD88. Satoh et al. also demonstrated that *Cutibacterium acnes* can induce IL-36γ through NF-κB in keratinocytes and subsequently IL-8, leading to cutaneous neutrophilia (77).

Fungal infection can induce IL-36 expression in epithelial cells and human PBMC (74, 78, 79). In oral candidiasis, IL-36α, β and γ transcript levels were all increased in the tongue of the sublingually challenged mice at 2 days post-injection (74). *Candida albicans (C. albicans)* infection resulted in increased IL-36 cytokines in human oral epithelial cells *via* NF-κB, MAPK and PI3K-dependent pathways (74). IL-36R-deficient mice were susceptible to acute oral candidiasis as evidenced by higher fungal loads and greater body weight loss, indicating the protective role of IL-36 in *C. albicans* infection (74).

### Lung

Influenza virus infection can trigger epithelial cell-derived IL-36 cytokines (22, 46, 80), which activated NF-κB signaling and increased inflammatory cytokines (e.g. IL-6 and IL-8) in the lung (46). However, the role of IL-36 in influenza virus infection is incompletely understood. Aoyagi et al. reported that IL-36R-deficient mice were protected from influenza virus-induced lung injury and mortality accompanied by reduced lymphocyte activation, accumulation of myeloid cells, pro-inflammatory cytokine and chemokine production (e.g., IL-6, IL-17, CXCL1, and CXCL10) and permeability of the alveolar-epithelial barrier (22). However, IL-36γ was upregulated in the lungs and played a protective role in severe H1N1 and H3N2 influenza infection *via* modulating macrophage polarization and activity (21). Lack of IL-36γ resulted in increased viral titers, higher levels of IL-6, and more severe pathology in the lungs (21). Interestingly, macrophages in IL-36γ-deficient mice exhibited an M2-like phenotype and were likely to undergo apoptosis by infection, whereas adoptive transfer of WT alveolar macrophages protected IL-36γ-deficient mice against influenza infection (21). The reason for the discrepancies from the studies using IL-36R- and IL-36γ-deficient mice are not known at present. Different animal models and interfering strategies, such as neutralizing antibodies, should be used to further confirm these results.

The role of IL-36 in *Mycobacterium tuberculosis* (*M. tuberculosis*) has been documented in several studies. *M. tuberculosis* infection induced IL-36γ expression in human macrophages *in vitro*, and in the lungs of infected mice *in vivo* (72, 81). Its expression was induced through microbial ligands, which triggered host TLR and MyD88-dependent pathways, and was further amplified by endogenous IL-1β and IL-18 (81). Increased IL-36γ transcriptional expression was also observed in the plasma and bronchoalveolar lavage (BAL) samples of patients with *Pseudomonas aeruginosa* (*P. aeruginosa*)- or *Streptococcus pneumoniae* (*S. pneumoniae*)-induced acute respiratory distress syndrome (ARDS) (23, 24). Animal studies revealed that IL-36 signaling pathway may play a protective role in the lung with bacterial infection. The induction of IL-36 contributed to antimicrobial peptide production and *M. tuberculosis* growth restriction through promoting the accumulation of Liver X Receptor and modulating cholesterol biosynthesis and efflux (82). Activation of autophagy in macrophages was considered another hallmark by IL-36γ in restricting *M. tuberculosis* growth (71). However, IL-36R deficiency showed negligible impact on *M. tuberculosis* infection in mice, as demonstrated by similar survival rates and bacterial loads (72). Additionally, IL-36γ-deficient mice were more susceptible to *S. pneumoniae* infection, as evidenced by increased mortality, ameliorated lung bacterial clearance and increased bacterial dissemination, which might be due to the reduced type-1 cytokine expression and impaired lung macrophage M1 polarization (23). Similarly, the protective effect of IL-36γ was also demonstrated in a *Klebsiella pneumoniae* (*K. pneumoniae*) mouse model (23). Interestingly, Sequeira et al. revealed that microbiota Bacteroidetes protected against *K. pneumoniae* colonization (83) *via* IL-36 signals and macrophages (83). In addition, administration of *Legionella pneumophila* to IL-36R-deficient mice resulted in more severe disease as evidenced by higher mortality, delayed lung bacterial clearance, increased bacterial dissemination to the spleen, and impaired innate immune responses compared to that in infected wild-type mice (73). In contrast, IL-36R^-/-^ and IL-36γ^-/-^, but not IL-36α^-/-^, mice were resistant to during *P. aeruginosa* infection, as demonstrated by the reduction of bacterial burden, pro-inflammatory cytokine production and lung injury. Further investigation is needed to determine the role of IL-36 in intracellular bacterial infection using various interfering methods, such as IL-36 cytokine knockout mice and neutralizing antibodies.

### Gut

Clinical evidence showed that ulcerative colitis patients had higher IL-36α in the colonic mucosa (36). Lack of IL-36R resulted in defective recovery following DSS-induced damage and impaired closure of colonic mucosal biopsy wounds due to the profound reduction of IL-22 (84). Interestingly, IL-36 can also regulate Treg/Th9 balance and the IL-23/IL-22 network in model of colitis induced by oxazolone, indicating that IL-36γ has multiple functions in modulating antigen-presenting cell function and in regulating T cell differentiation in a mouse model (58, 59). Russel et al. reported that infection with *Citrobacter rodentium* resulted in reduced CD11b^+^F4/80^+^Gr-1^+^ inflammatory cell recruitment, imbalanced Th1/Th17 responses and increased bacterial colonization of the colon in IL-36R^-/-^ mice (36). Accordingly, suppressed Th17, but enhanced Th1 differentiation was observed *in vitro* by IL-36α supplement (36, 57). However, since IL-36 is necessary for IL-22 production in DSS-induced colitis, it is not clear whether IL-36 differently regulates Th17 and Th22 differentiation *in vivo* among various animal models of gastrointestinal dysregulation.

### Other Organs

Although IL-36 has been detected in hepatocytes (12, 63), the function of IL-36 in the liver remains unclear. Higher levels of IL-36α were observed in chronic hepatitis B virus (HBV) patients compared with that in healthy individuals (85). The positive correlation between IL-36α and HBV-DNA titers may indicate the potential involvement of IL-36 in antiviral immunity during chronic infection (85). Additionally, hepatitis C virus infection significantly increased the production of IL-36Ra but not IL-36 agonist ligands in human monocytes, leading to reduced NK cell activation (86). Further research is needed to dissect the role of IL-36 in liver resident cells (e.g., kupffer cells, hepatic stellate cells and sinusoidal endothelial cells) as well as in different liver disease models.

In addition to lung infections, IL-36α and IL-36γ were also upregulated in the mouse cornea in early responses to *P. aeruginosa* challenge (70). Exogenous IL-36γ treatment enhanced corneal innate immunity and alleviated *P. aeruginosa* keratitis. The protective role of IL-36γ required S100A9 and was partially dependent on the CXCL10/CXCR3 axis (70). On the contrary, IL-36Ra treatment exacerbated the outcome of *P. aeruginosa* keratitis (70).

Louis et al. reported that a truncated IL-36γ-encoded plasmid can act as a potent adjuvant for a DNA-encoded Zika virus (ZIKV) vaccine. Immunization with truncated IL-36γ promoted antiviral T cell responses and protected mice from ZIKV challenge (68). Moreover, co-delivery of truncated IL-36γ can also enhance antiviral immunity against HIV and influenza DNA vaccines (68). Besides, both *in vivo* and *in vitro* studies have proved that IL-36 treatment reduced HSV-2 replication in a lethal genital infection model and in human vaginal epithelial cells (66, 67). The absence of IL-36γ led to reduced mature neutrophil recruitment to the vaginal microenvironment at early times in HSV-2 infection (66). These findings set the stage for IL-36 in infectious diseases and shed light on IL-36 in the next generation of vaccines.

## IL-36 as a Therapeutic Target of COVID-19

Although several vaccines have been issued for the emergency use authorization for the prevention of coronavirus disease 2019 (COVID-19), intensive efforts are underway to investigate the immunopathology of this infectious disease caused by severe acute respiratory syndrome coronavirus 2 (SARS-CoV-2). The majority of patients with COVID-19 are asymptomatic or mild flu symptoms, but in some individuals, who are critically ill with COVID-19, it can develop into severe pneumonia and life-threating ARDS. The members of IL-1 family including IL-1β and IL-33 may contribute to the inflammation and antiviral immune regulation in COVID-19. In severe cases of COVID-19 patients, increased IL-1α and IL-1β have been detected (87, 88). SARS2-CoV-2 may facilitate IL-1β activation and maturation, leading to the cytokine storm together with other pro-inflammatory mediators such as IL-6 and TNF-α (89). Blockage of IL-1 signals using IL-1 receptor antagonist Anakinra might be associated with clinical improvement in patients (87, 88). The alarmin cytokine IL-33 may also play a detrimental role in severe COVID-19 cases through expanding the pathogenic T cells, inducing hyperinflammation, and promoting the pro-fibrotic type 2 innate immune cells (90).

In patients with COVID-19, airway epithelial cells showed an average three-fold increase in expression of the SARS-CoV-2 entry receptor angiotensin-converting enzyme-2 ACE2 (91). Notably, bronchial epithelial cell ACE2 expression was correlated with IL-36β in bronchoalveolar lavage in asthma cohorts (92). Moreover, human basal lung epithelial cells exposed to poly(I:C) exhibited significant increase in protein concentrations of IL-36γ (55). SARS-CoV-2 viral RNA and viral nucleocapsid protein can be detected in gastrointestinal tissues from the patients (93, 94). This might be due to the highly expressed ACE2 in human gastrointestinal epithelial cells (95). IL-36γ was predominantly detected in human intestinal epithelium (44), and induced expression of chemokines, GM-CSF and IL-6 (44). Therefore, IL-36 may contribute to the ACE2 regulation and intestinal inflammation in COVID-19 patients. In addition, vasculopathy and lymphoid infiltrate of the superficial and deep dermis is main cutaneous manifestations in COVID-19 patient (96–99). It was reported that ACE2 and SARS-CoV-2 RNA can be detected in the blood vessels (100, 101), whereas IL-36γ and IL-36R also expressed in human dermal microvascular endothelial cells (HDMEC) (18, 102, 103). It is likely that SARS-CoV-2 infection in endothelia cells may induce IL-36 secretion, leading to leukocytes infiltration and skin symptoms in COVID-19 patients. Furthermore, high expression of ACE2 was also found in keratinocytes (104), which can increase IL-36 expression by Poly I:C stimuli (80, 105). Besides, IL-36 upregulated ACE2 expression in human keratinocytes according to publicly available RNAseq data (106). SARS-CoV-2 infection may promote IL-36 production from keratinocytes and exacerbate skin lesion. These findings suggest that IL-36 might be a potential biomarker of disease severity in COVID-19.

Profound pulmonary infiltration of myeloid cells including neutrophils and macrophages/monocytes have been found in COVID-19 patients with severe clinical progression (107–109). Local IL-36 may drive these myeloid cell recruitment and activation, resulting in pulmonary hyper-inflammation (37, 41, 55). Moreover, infiltrated neutrophils may produce high concentrations of neutrophil extracellular traps (NETs) (110–112), and induce lung epithelial cell death in COVID-19 patients (110). In addition, IL-36 can induce IL-6 and IL-8 expression and further increase inflammatory responses (3), while IL-1β and IL-6 are capable of inducing IL-36 expression (81). This proinflammatory positive loop may also contribute to immunopathogenesis of COVID-19. IL-36 was upregulated in the lungs after influenza virus infection (22, 46), and led to inflammatory cytokines production (22). GM-CSF, which is rapidly produced by pathogenic Th1 cells in COVID-19, can act with other inflammatory cytokines to form a cascade signature of inflammatory monocytes with high IL-6 expression (113). Importantly, IL-36 increases the secretion of GM-CSF by activation of Erk1/2, MAPK and JNK (3). IL-36γ cooperated with poly(I:C) in human macrophages also promoted GM-CSF expression (55). These findings indicate that IL-36 may contribute to the induction of IL-6-producing monocytes through GM-CSF. Moreover, IL-36, as a strong inducer of murine Th1 cells (14, 57), may play a role in human Th1 differentiation (56), and exacerbate lung pathogenesis by enhancing pathogenic Th1 responses and the following cytokine storm. In addition to Th1 responses, IL-36 is also a key regulator in IL-17 responses through regulating not only adaptive Th cells, but also γδ T cells and ILC3 (34, 69). Notably, elevated IL-17 levels have been reported in patients infected with coronavirus, including SARS-CoV, MERS and SAR-CoV-2 (89). Blockage of IL-36 signals may lead to proposals for a therapeutic approach to COVID-19 through modulating proinflammatory IL-17 responses.

**Figure 2:**
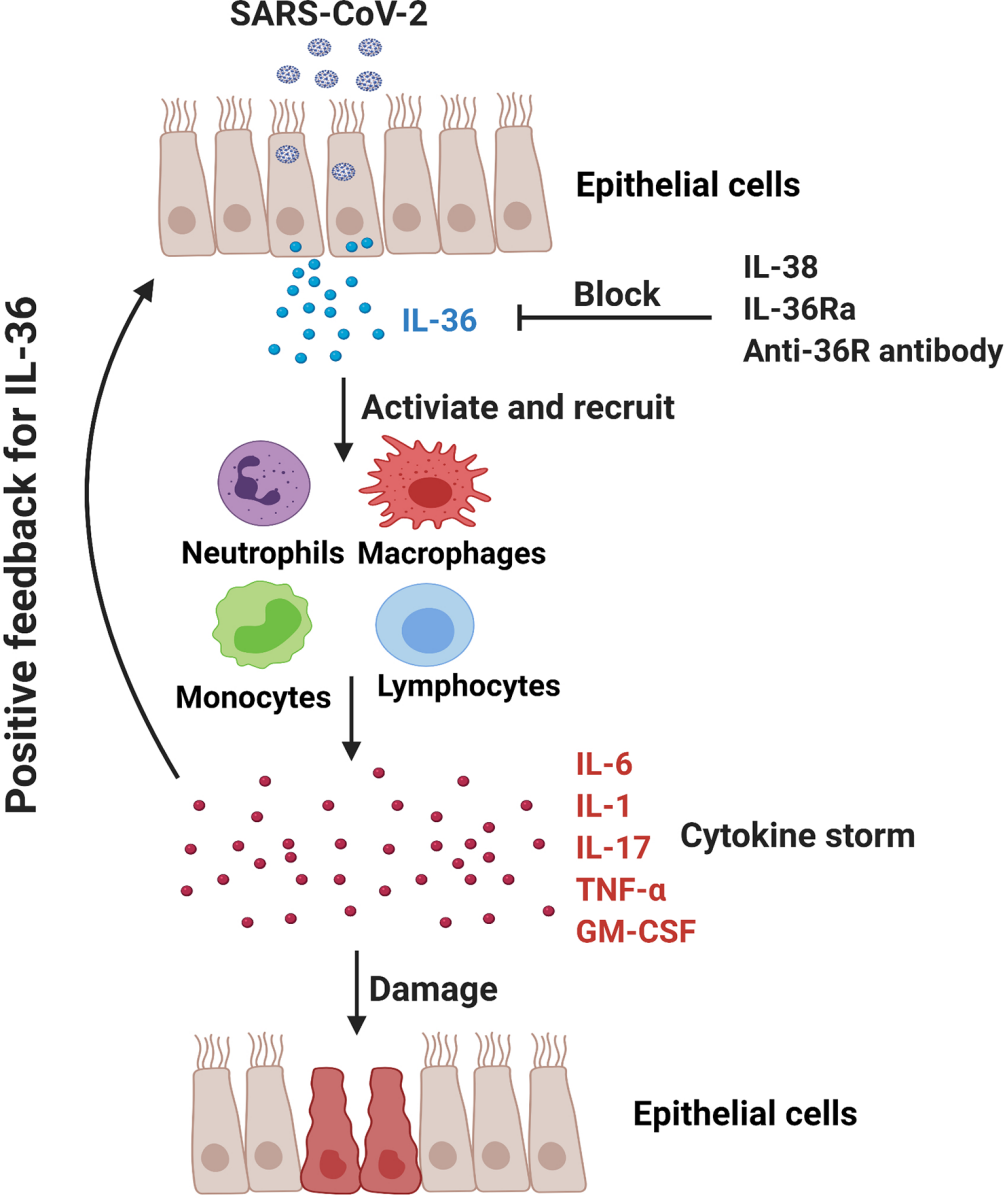
In COVID-19 patients, SARS-CoV-2 may promote hyperinflammation in the lung and exacerbate tissue damage. IL-36-activated inflammatory immune cells (e.g., monocytes, macrophages, neutrophils and pathogenic T cells) produce IL-6, IL-1, IL-17, TNF-α and GM-CSF to further amplify IL-36 responses. IL-36Ra and IL-38, as the natural antagonistic mediators in IL-36 family might be a promising therapeutic target for COVID-19 *via* inhibiting IL-36 signaling pathway and alleviating pulmonary hyperinflammation.

The application of IL-1 receptor antagonist Anakinra has shown the potential therapeutic effect in COVID-19 patients (87, 88, 114). In addition to the agonistic ligands, IL-36Ra acts as an antagonist for IL-36 signaling pathway and may reduce IL-36-driven inflammation *via* competing with their receptor IL-36R. Additionally, IL-38, the newest member of IL-36 family, can downregulate poly (I:C)-induced IL-6, CCL5, and IL-1β expressions in bronchial epithelial cells, indicating the anti-inflammatory role of IL-38 in viral infection (115). Notably, it is reported that IL-38 increased significantly in influenza and COVID-19 patients and may function as a suppressor cytokine that inhibits IL-1, IL-6 and TNF-α in COVID patients (116, 117). Significant efforts are undergoing to develop neutralizing antibody targeting the IL-36R signaling axis for the therapy of IL-36-mediated diseases. Antibody-mediated blockade of IL-36R signaling reverses established fibrosis in chronic intestinal inflammation in mice (118) Chimeric antibodies MAB92 and MAB04, binding primarily to domain-2 of the human and mouse IL-36R proteins respectively, have been demonstrated to inhibit skin inflammation (69, 119). Anti-mouse IL-36R mAb M616 specific for murine IL-36R is also under experimental trials (120). Importantly, a single dose of BI 655130, a monoclonal antibody against the IL-36 receptor, reduced the severity of generalized pustular psoriasis in patients (7). Therefore, application of IL-36Ra, IL-38 and IL-36R mAbs might be a promising therapeutic way in COVID-19 patients *via* inhibiting IL-36-mediated hyperinflammation (**Figure 2**).

## Conclusions and Perspectives

The accumulated evidence during the past decade indicates that IL-36 plays a fascinating role in systemic inflammatory diseases and cancer (36, 51, 121). The genetic deficiency of IL-36Ra leads to generalized pustular psoriasis (GPP), while IL-36Ra was considered an effective treatment of psoriasis diseases (106). Inhibition of IL-36R with a single dose of BI 655130 monoclonal antibody reduced the severity of GPP in patients (7). Interestingly, direct intra-tumoral delivery of IL-36 mRNA led to robust anticancer responses in a broad range of tumor microenvironments (122). These studies highlight the clinical therapeutic potential of IL-36 in inflammatory diseases and cancer.

Several aspects of IL-36 are less understood and remain somewhat controversial. It is still not clear what the distribution of IL-36R is in immune cells, especially in humans (41, 42). Whether the receptor is inducible by other host factors or pathogens is still not well understood.

IL-36 may be more than just a general inflammatory marker but a pathogenic sensor due to its location at epithelial/environmental interface and its release and activation by pathogenic damage (123). It is less well elucidated what the crucial bioactive forms of IL-36 *in vivo* are or how they are generated in each infectious disease condition. Moreover, it is striking that the different isoforms of IL-36 are expressed differently under physiological as well as pathological conditions, and have different functions in the development of infection. Further investigations are needed to elucidate the molecular mechanisms underlying their biological functions, especially in COVID-19. In terms of clinical implications, future study of the functions of the IL-36/IL-36R pathway in disease pathogenesis may facilitate the development of therapeutics targeting these cytokines for the treatment of infectious diseases.

## Author Contributions

XW wrote the manuscript. PY and YL wrote and critically revised the manuscript. All authors contributed to the article and approved the submitted version.

## Funding

This work was supported by grants from the National Natural Science Foundation of China 81800506 and Natural Science Foundation of Hunan Province of China 2019JJ40494 to PY. The NIH AI153586 and the UTMB IHII Data Acquisition Grant to YL.

## Conflict of Interest

The authors declare that the research was conducted in the absence of any commercial or financial relationships that could be construed as a potential conflict of interest.
